# Cascade and Fusion: A Deep Learning Approach for Camouflaged Object Sensing

**DOI:** 10.3390/s21165455

**Published:** 2021-08-13

**Authors:** Kaihong Huang, Chunshu Li, Jiaqi Zhang, Beilun Wang

**Affiliations:** 1 Department of Computer Science and Engineering, Southeast University, Nanjing 211189, China; huangkaihong@seu.edu.cn; 2 Department of Artificial Intelligence, Southeast University, Nanjing 211189, China; chunshu@seu.edu.cn; 3 Department of Computer Science, Brown University, Providence, RI 02860, USA; jiaqi_zhang2@brown.edu

**Keywords:** optical sensor, deep learning, camouflaged object, object detection, edge computing

## Abstract

The demand for the sensor-based detection of camouflage objects widely exists in biological research, remote sensing, and military applications. However, the performance of traditional object detection algorithms is limited, as they are incapable of extracting informative parts from low signal-to-noise ratio features. To address this problem, we propose Camouflaged Object Detection with Cascade and Feedback Fusion (CODCEF), a deep learning framework based on an RGB optical sensor that leverages a cascaded structure with Feedback Partial Decoders (FPD) instead of a traditional encoder–decoder structure. Through a selective fusion strategy and feedback loop, FPD reduces the loss of information and the interference of noises in the process of feature interweaving. Furthermore, we introduce Pixel Perception Fusion (PPF) loss, which aims to pay more attention to local pixels that might become the edges of an object. Experimental results on an edge device show that CODCEF achieved competitive results compared with 10 state-of-the-art methods.

## 1. Introduction

Object detection as a fundamental component of optical sensor systems has been extensively applied in various practical scenarios, such as automatic driving, human–computer interactions, and industrial production. However, when practitioners try to apply object detection techniques in biological, security, or military scenarios, traditional object detection algorithms are often incapable of dealing with harsh or extreme situations that are even challenging to the naked eye. A typical example is to identify species with camouflage capabilities from images acquired by non-invasive sensors (namely, camera traps).

**Figure 1:**
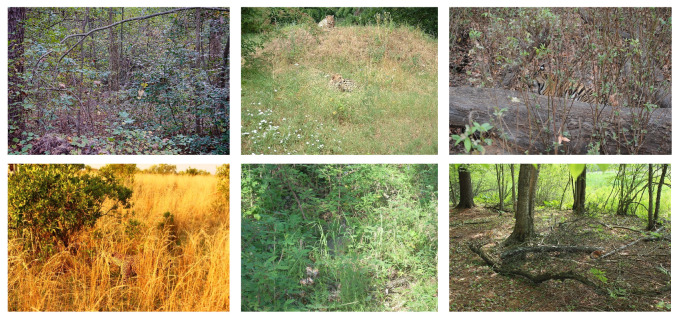
Images of camouflaged animals. These camouflaged creatures used to deceive the visual system pose a new challenge to the algorithm.

Traditional animal detection algorithms for fixed-point sensors rely on additional motion perception hardware and assume that the appearance of the creature has a certain degree of saliency [1,2]. However, we observed that, limited by the imaging quality of the sensor and the illuminance conditions, the animals often showed similarities in color and texture with the background. Figure 1 shows several examples of images of camouflaged animals. This brings about the need for a powerful detection method for camouflage targets. This challenging task is named *camouflaged object detection* (COD).

**Figure 2:**
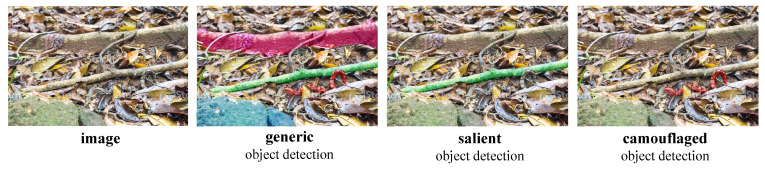
An example to show the difference between generic target detection (GOD), salient target detection (SOD), and camouflaged target detection (COD). GOD detects different objects in the image and labels their categories. SOD detects targets that grab human attention, whilst COD aims to detect objects with similar patterns to the background. For simplicity, many objects, such as leaves and branches, are not marked in generic object detection.

COD aims to estimate the region of an object that is concealed in its surroundings at the pixel level. Known as *camouflage* in the biological literature, the phenomenon of visual concealment exists extensively in both natural and artificial objects [3]. As shown in Figure 2, different from salient object detection (SOD), i.e., detecting objects of potential human interest, COD focuses on targets that are less likely to capture human attention or attempt to deceive visual perception systems in an adversarial manner. In early studies, COD was often approached as foreground detection, which utilizes the hand-crafted features computed by edges, brightness, corner points, texture, or temporal information [4] to separate the camouflaged object and the background [5,6,7]. However, the hand-crafted features are incapable of detecting all the sophisticated camouflage strategies in the real application scenarios.

Recently, the unprecedented success of deep neural networks, particularly convolutional neural networks (CNNs) [8], have benefited various fields of computer vision, including image classification [8,9,10,11,12], image generation [10,13], and generic object detection [14,15,16,17]. Despite the wide variety of CNN-based object detection models, special designs are necessary to build models for COD. On one hand, generic object detection (GOD) detects targets with bounding boxes, rather than pixel-level segmentation; moreover, the segmentation in COD is based not on semantics, but on saliency from the human perspective, which is not modeled in GOD models. On the other hand, models that are designed for salient object detection are not competent to accurately detect camouflaged objects. Although SOD models do non-semantic segmentation and model saliency, they do not specialize in finding the vague boundaries of objects, as salient objects tend to be visually distinct from the surroundings.

Researchers have proposed several feasible methods for COD. ANet uses an additional classification networks to refine the prediction results of traditional target segmentation networks [18]. However, its two-stream structure is still based on the traditional convolutional network structure and, thus, cannot provide the perceptual ability required by the COD task. RankNet [19] takes another approach and generates saliency prediction by instance-level ranking-based region. SINet utilizes a cascaded network, which divides the network into a Search Module (SM) and an Identification Module (IM), to hierarchically refine the prediction map [20].

However, current methods still have difficulty in accurately estimating the detection map. Specifically, the remanent challenges lie in the attacks of low signal-to-noise ratio features in the decoding process. Generally, object detection models consist of an encoder to extract features and a decoder to fuse features [21]. The output features of the shallow encoder layers have a low signal-to-noise ratio due to the lack of semantic orientation [22]. Fortunately, by using a specially designed network called decoder, we can combine them with semantic information extracted by subsequent convolutional layers to obtain acceptable prediction maps. However, biological studies have shown that camouflaged targets will produce more noisy interference on the visual perception system [3,23,24]. Without precise control of the feature interweaving process, the decoder is vulnerable to attacks of significantly larger background noise, which leads to vague target boundaries and misjudgment in extreme situations.

To address the problem, we propose a novel COD framework, CODCEF (Camouflaged Object Detection with Cascade and FEedback Fusion). Evidence [20,25,26] has shown that dividing the overall task into multiple sub-tasks is a viable approach. Therefore, CODCEF uses two cascaded network components, the Wide Attention Component (WAC) and the Accurate Detection Component (ADC). Compared to a single encoder–decoder structure, the cascaded structure can effectively suppress the residual noise in the decoding process. Based on cross feature modules (CFMs) [27], which selectively fuse low-level and high-level features, we designed the Feedback Partial Decoders (FPDs) to serve as decoders in both components.

Compared with traditional decoders based on addition and concatenation, the FPD can better tolerate low signal-to-noise ratio features by using the feedback-based structure with multi sub-decoders. In addition, we observe that the loss function for the local region can effectively improve the generalization ability of the model [27]. Following this observation, we propose a loss function, called Pixel Perception Fusion Loss (PPF). PPF gives additional weight to the sharply changing pixels that may become the segmentation boundary on the basis of the binary cross entropy and intersection-over-union [28]. Compared to 10 state-of-the-art methods for SOD and COD, our method demonstrated competitiveness in prediction accuracy on the three COD benchmark datasets. In summary, the paper makes the following contributions:

- New framework: We propose CODCEF, a new framework for camouflaged object detection. With the cascaded structure and the Feedback Partial Decoders, CODCEF is endowed with superior noise suppression capabilities required for camouflaged target detection, even on a shallow backbone network (ResNet-50).
- Efficient loss function: We propose a new loss function, namely the Pixel Perception Fusion (PPF) loss, to train the model. The PPF loss fits the characteristics of the cascaded structure, makes the model pay further attention to the high-frequency local pixels and facilitates the training of the model.
- Experimental evaluation: On an Nvidia Jetson Nano, we compare CODCEF with 10 state-of-the-art COD or SOD models on three datasets, including COD10K, CAMO, and CHAMELEON. The experimental results show that CODCEF demonstrates stable and accurate camouflage target recognition capabilities. Simultaneously, with the use of additional cameras and portable power supplies, we proved the feasibility of the model on portable edge devices in a real environment. The source code will be publicly available at https://github.com/HHHKKKHHH/CODCEF (accessed on 20 May 2021).

The rest of this paper is organized as follows. Section 3 briefly introduces the motivation and discusses details the proposed framework. Section 4 and Section 5 reports the experimental results and the ablation study. Finally, Section 6 draws our conclusions.

## 2. Related Work

In recent years, researchers have made outstanding contributions to the field of object detection using deep learning methods. In this section, we review the related work of the three major tasks of object detection: generic object detection, salient object detection, and camouflaged object detection.

### 2.1. Generic Object Detection (GOD)

GOD is an important and fundamental branch of target detection, which generally pursues semantic segmentation or classification. Exiting GOD models can be grouped into two categories: two-stage detection and one-stage detection, where the former frames the detection as a progressive process, while the later frames it to “complete in one step”.

In 2014, Girshick et al. proposed RCNN [29], a simple and scalable two-stage detection model by selective search. SPPNet et al. enables CNNs to generate a fixed-length representation regardless of the size of image of interest without rescaling the image [30]. Various types of enhanced RCNN, such as FastRCNN [31], FasterRCNN [32], and MaskRCNN [33], have made significant progress in efficiency and prediction accuracy. On the basis of Faster RCNN, FPN exploited the inherent multi-scale, pyramidal hierarchy of deep convolutional networks to construct feature pyramids with a marginal extra cost [34].

YOLO, a first one-stage detector, was proposed by R. Joseph et al. Later, R. Joseph made a series of improvements on the basis of YOLO [35,36,37]. RetinaNet focuses on hard, misclassified examples during training [38].

### 2.2. Salient Object Detection (SOD)

SOD aims to localize the regions of an image that attract human attention. Before the deep learning revolution, conventional salient object detection models used handcrafted features, which utilized the contrast between pixels [39,40] to define saliency, whose generalization and effectiveness were limited. Existing SOD deep learning networks [41,42,43,43] mainly focus on designing an effective decoder to achieve high–low level feature aggregation.

The early deep learning methods [44,45] transfer to generate a high-dimensional feature space and create a saliency map. On the basis of the traditional encoder–decoder structure, Wu et al. [25] abandoned the low-level features and designed a cascade partial decoder with finer detailed information. Instead of using a backbone network, Liu et al. [46] mimicked the human visual perception system and proposed a general small sensing network that can be used for rapid detection. PFANet improves on the traditional pyramid network structure and introduces a channel-wise attention (CA) model and spatial attention (SA) model [22].

EGNet leverages the salient edge features to help the salient object features locate objects [47]. Pang et al. [48] integrated the information of adjacent layers and integrated multi-scale information to retain the internal consistency of each category. $F^{3}\mathrm{Net}$ introduces a Cross Feature Module (CFM) for the adaptive selection of complementary information when aggregating multi-scale features [27]. However, simply stacking decoders composed of CFM will cause accuracy degradation due to the network depth, while our cascaded structure has the ability to accommodate more decoders.

### 2.3. Camouflaged Object Detection (COD)

COD aims to discover objects that are deliberately hidden in the image.

#### 2.3.1. Datasets

The Chameleon dataset [49], which contains 78 images of camouflaged objects, was first published but is not enough to support the training and testing of neural networks. CAMO dataset [18], which includes 1250 camouflaged images divided into eight categories, laid a foundation for subsequent research in COD. Fan et al. [20] provided a more comprehensive dataset, named COD10K. They released 3040 camouflaged images for training and 2026 images for testing.

#### 2.3.2. Methods

In early studies, most researchers used low-level features including texture, edge, brightness, and color to discriminate objects [5]. Zhang et al. [6] compensated for the lack of static features by disguising the movement information of the camouflaged object. TGWV [7] uses a texture-guided weighted voting method that can efficiently detect foreground objects in camouflaged scenes. However, these manual features are vulnerable to attacks from the sophisticated camouflage strategies. Therefore, recent studies have turned to deep learning to incorporate more information and features.

Among those, Le et al. employed an auxiliary classification network to predict the probability of containing a camouflaged object in an image. Ren et al. [6] formulated texture-aware refinement modules emphasizing the difference between the texture-aware features. Dong et al. [7] used a significant large receptive field to provide rich context information and an effective fusion strategy to aggregate features with different levels of representation. RankNet [19] used the localization model to find the discriminative regions and the segmentation model to segment the full scope of the camouflaged objects.

MGl [50] uses a novel Mutual Graph Learning model, which generalizes the idea of conventional mutual learning from regular grids to the graph domain. SINet [20] uses a Search Module (SM) and an Identification Module (IM) to hierarchically refine the prediction map and use PDC [25] as decoders. However, PDC, which mixes features by addition and concatenation is not robust enough to deal with low signal-to-noise ratio features. Therefore, we introduce decoders with a selective fusion strategy to prevent features from being contaminated during the fusion process.

## 3. Materials and Methods

In this section, we describe the details of the proposed framework, the Camouflaged Object Detection with Cascade and FEedback Fusion (CODCEF) and the corresponding optimization strategy.

### 3.1. Overview

CODCEF is composed of two cascaded network components, the Wide Attention Component (WAC) to obtain an approximation of the detected outline and the Accurate Detection Component (ADC) to refine the edge of previous prediction and eliminate residual noise. Although the two components are very similar in structure, different contexts make their targets significantly different. WAC as a relatively independent module, takes, as input, the original RGB image and outputs a prediction that can be used to calculate loss of network. The ADC combines the output of results with the middle-level features of the original image to screen out possible misleading information and noise.

In each component, we use Feedback Partial Decoder (FPD) based on Cross feature module (CFM) [27]. Though selective feature interleaving and a feedback loop, FPD with multiple sub-decoders can fully utilize the structural details and semantic information in the multi-level features. Dividing the model into multiple parts with clear responsibilities allows us to capture the periodic evaluation results of the model from its intermediate results, which leads to a more objective and comprehensive loss function, Pixel Perception Fusion Loss (PPF). PPF gives extra weight to sharply changing pixels to focus the attention of framwork on possible object boundaries.

**Figure 3:**
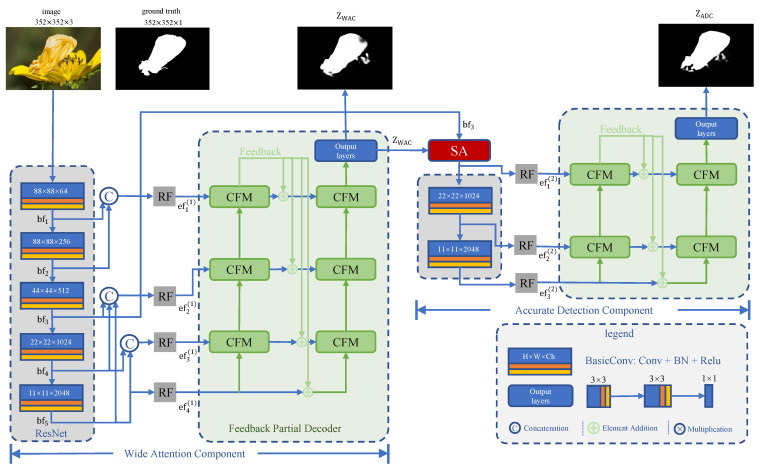
Overview of the CODCEF framework. The WAC and ADC generate two stage prediction maps, and $Z_{\mathrm{ADC}}$ is the final result of the network. The RF is the receptive field module, which is shown in Figure 4. The SA is the search attention function [25]. The CFM is the cross feature module, which receives high-level features from the green arrow and low-level features from the blue arrow shown in Figure 5.

The structure of the proposed model is shown in Figure 3.

### 3.2. Wide Attention Component

In the WAC, for an RGB image $I\in R^{W\times H\times 3}$, we use ResNet-50 [9] with the resolutions $(\frac{H}{k},\frac{W}{k}),\;k=4,4,8,16,32$, to extract basic features at different levels denoted as $bf_{i}\in R^{\frac{W}{k}\times \frac{H}{k}\times c_{i}}$, where $c_{i}$ is the channel number of the *i*-th ResNet, i=1,⋯,5. ResNet is a pre-trained deep residual backbone network. It uses the residual mechanism to effectively improve the accuracy degradation caused by the depth of the network. Considering the overall design of the model, we choose the ResNet-50 pre-trained model as the encoder of CODCEF. According to the evidence from [9,34], basic features can be divided into low-level ($bf_{1}$ and $bf_{2}$) with more resolution information, mid-level $bf_{3}$, high-level ($bf_{4}$ and $bf_{5}$) with more semantic information.

To save the characteristic information for the decoder, we use up-sampling and down-sampling operations to normalize the resolution of the basic features to the maximum resolution in each binding unit and cascade the proximity features, obtaining four hybrid features.

**Figure 4:**
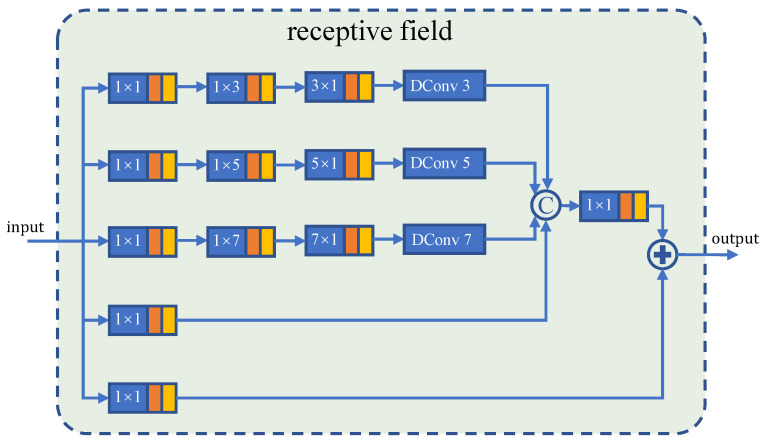
An illustration of a modified receptive field [20]. Dconv is short for dilation convolution. The sizes of the convolutional kernels are marked on the convolution layers.

Due to the challenge of the COD task, we required a stronger sense of the local features. However, considering the gradient calculation of the model, suddenly deepening the model would bring devastating consequences to the training. According to [46], receptive fields block module (RFB), which combines multiple branches with different kernels, and dilated convolution layers can reduce some loss in the feature discriminability as well as robustness. Thus, in order to further enhance the identification features without over-deepening the network, we use the modified RF module shown in Figure 4 to transform hybrid features into enhanced features [20]. Specifically, enhanced features denoted as {$ef_{i}^{\left(1\right)}\;|\;i=1,2,3,4$} are given by

$$\begin{array}{cc} \; & ef_{1}^{\left(1\right)}={\mathrm{RF}}_{1}\left({\mathrm{DOWN}}_{2}\left(bf_{1}+bf_{2}\right)\right)\\ \; & ef_{2}^{\left(1\right)}={\mathrm{RF}}_{2}\left(bf_{3}+{\mathrm{UP}}_{2}\left(bf_{4}\right)+{\mathrm{UP}}_{4}\left(bf_{5}\right)\right)\\ \; & ef_{3}^{\left(1\right)}={\mathrm{RF}}_{3}\left(bf_{4}+{\mathrm{UP}}_{2}\left(bf_{5}\right)\right)\\ \; & ef_{4}^{\left(1\right)}={\mathrm{RF}}_{4}\left(bf_{4}\right) \end{array}$$

where {${\mathrm{RF}}_{i}\;|\;i=1,2,3,4$} are the modified receptive fields shown at Figure 4. ${\mathrm{DOWN}}_{k}$ or ${\mathrm{UP}}_{k}$ means down-sampling or up-sampling by multiples of *k*.

After obtaining a set of $ef_{i}^{\left(1\right)}$, we used a Feedback Partial Decoder (**FPD**, see Section 3.4) with three feedback loops to interweave and merge features into a phased result, denoted as $Z_{\mathrm{WAC}}$ shown in Figure 3 top-middle.

### 3.3. Accurate Detection Component

Since the network has two main components, we required a function to summarize the prediction results of the front component without excessively increasing the network complexity. This motivated us to use a Search Attention function (SA) [22] to multiply a preliminary prediction by the middle-level feature $bf_{3}$, which contains most of the features of the original image with low noise, generating the attention map *A*. In addition, to prevent the existing results $Z_{\mathrm{WAC}}$ from excessively restricting subsequent perception, we used a Gaussian filter to actively blur the boundary. Specifically, *A* is given by:

(1) $$A=F_{max}\left(G\left(Z_{\mathrm{WAC}}\right),Z_{\mathrm{WAC}}\right)⊙bf_{3}$$

where G(·) is a typical Gaussian filter with standard deviation σ=32 and kernel size λ=4. ⊙ denotes elements-wise multiplication. $F_{max}$ is an elements-wise maximum function. Equation (1) aims at highlighting salient regions in $Z_{\mathrm{WAC}}$, which prevents them from being overly blurred after Gaussian filtering.

Next, *A* goes through a shallow convolutional network to extract certain features, as shown in Figure 3. These features can be enhanced by modifying the receptive fields as shown in Figure 4 to obtain $\left\{ef_{i}^{\left(2\right)}\;|\;i=1,\cdots ,3\right\}$.

To holistically obtain the final prediction map, we further utilized the FPD (discussed in Section 3.4). Unlike in WAC, we only set up two layers of feedback loops for the FPD in ADC. Specifically, the final prediction map $Z_{\mathrm{ADC}}$, shown at Figure 3 top-right, is given by:

(2) $$Z_{\mathrm{ADC}}={\mathrm{FPD}}_{2}\left(ef_{1}^{\left(2\right)},ef_{2}^{\left(2\right)},ef_{3}^{\left(2\right)}\right)$$

where ${\mathrm{FPD}}_{n}$ means a feedback partial decoder with *n* feedback loops.

### 3.4. Feedback Partial Decoder

Unlike in SOD, the significant regions in COD are more complex. More specifically, the low-level features have a low signal-to-noise ratio bought by similar background elements and vague boundaries of high-level features, which leads to the less clear semantic information.

**Figure 5:**
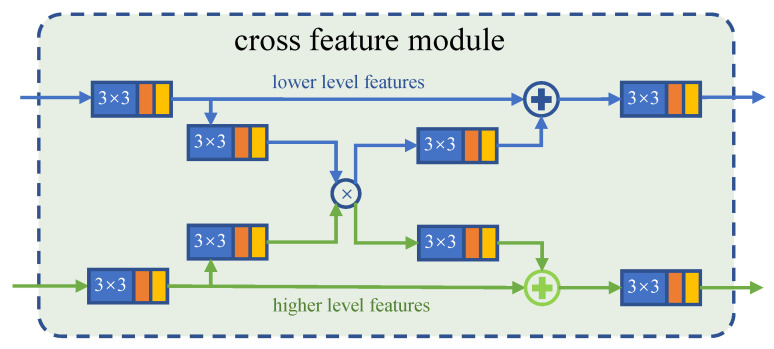
Framework of the cross feature module [27]. The N×M marked on the convolutional layer indicates the size of the convolution kernel. The other legend is the same as Figure 3.

This motivated us to use a cross feature module (CFM) [27], as shown in Figure 5, to build the Feedback Partial Decoder (FPD). CFM receives both low-level features and high-level features and makes full use of the extracted boundary information and semantic information through selective feature interleaving. In CFM, high-level features and low-level features cross each other by element-wise multiplication, which is effective in suppressing the background noise of the feature and sharpening the boundary of the prediction map.

Although we can obtain a clear map of a camouflaged object by cascading a series of CFM, some precise boundary features will be ignored in multiple feature aggregation. The FPD has two parallel sub-decoders, each of which is composed of several CFMs connected in series. In traditional decoders, multiple network layers are usually connected in parallel to supplement the missing information [21,25], which brings about instability and complexity. Thus, we further feedback refined results that are already enhanced by several CFMs into a second sub-decoder.

The main result of the first sub-decode, as supplementary information, will be fed back into input streams of the second one. This allows us to effectively suppress the high background noise caused by the confusing target in the shallow network. The output of the second information path is integrated through the information of the three-layer convolutional network to obtain a single-channel saliency prediction map. The whole process of FPD can be formulated as Algorithm Section 3.4, where $Ds_{i}\left(\cdot \right)$ means the downsampling operation, $Cr_{i}^{j}\left(\cdot \right)$ is the *i*-th CFM in *j*-th sub-decoder, and Output(·) is the output laryers shown in Figure 3.

The experiment in [27] shows that F3Net, which also uses decoders composed of CFM, becomes degraded when using more than two sub-decoders, while using the cascaded structure, our method can give full play to the feature fusion capabilities of the four sub-decoders.

**Algorithm 1:** Feedback partial decoder.

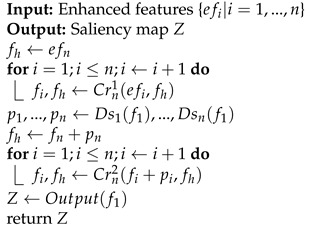



### 3.5. Pixel Perception Fusion Loss

Traditional image segmentation loss functions, such as binary cross entropy and intersection-over-union [28], can objectively evaluate the prediction map of the model in the local structure as well as the global structure. However, in view of the particularity of the COD task, we focused more on pixels with sharp changes in the gray value. The camouflaged object often has a slight grayscale mutation at the edge relative to the background, which generally comes from the difference of shadow or color convergence [23].

In order to use this edge information, we introduce Pixel Perception Fusion Loss (PPF), which consists of Pixel Frequency Aware Loss (PFA) [27] to optimize the prediction results of each component. PFA consists of two parts, a weighted binary cross entropy (wBCE) and a weighted intersection-over-union(wIoU), both of which give more weight to the high frequency parts of the image compared with the basic BCE and IoU. Mathematically, this additional weight for pixels in (i,j) denoted as $w_{i,j}$ is given by:

(3) $$w_{i,j}=\gamma \left|\frac{\sum_{\left(x,y\right)\in A_{i,j}^{k}}gt_{x,y}}{\left|A_{i,j}^{k}\right|}-gt_{i,j}\right|$$

where $A_{i,j}^{k}=\left\{\left(x,y\right)\;|\;\sqrt{{\left(x-i\right)}^{2}+{\left(y-j\right)}^{2}}\leq k\right\}$, and gt is the ground truth of this image. In fact, (3) is equivalent to a convolution with a kernel size of 2×k and k-padding. Specifically, in CODCEF, k=15 and γ=5. Clearly, for the local area where the gray value changes drastically in the picture, $w_{i,j}$ will be larger, which leads to more significant and targeted prediction loss assessment.

Thus, wBCE are computed by:

(4) $$L_{\mathrm{wBCE}}=-\frac{\sum_{i=1}^{H}\sum_{j=1}^{W}\left(1+\gamma w_{i,j}\right)\log\mathrm{Pr}\left(p_{i,j}=gt_{i,j}|\psi \right)}{\sum_{i=1}^{H}\sum_{j=1}^{W}\gamma w_{i,j}}$$

where $p_{i,j}$ is the point (i,j) of the prediction from ADC or WAC and $\mathrm{Pr}(p_{i,j}=gt_{i,j}\;|\;\psi )$ is under the current network parameters ψ—the probability that the predicted map is equal to ground truth.

wIoU are computed by:

(5) $$L_{\mathrm{wIoU}}=1-\frac{\sum_{i=1}^{H}\sum_{j=1}^{W}\left(gt_{i,j}\times p_{i,j}\right)\times \left(1+\gamma w_{i,j}\right)}{\sum_{i=1}^{H}\sum_{j=1}^{W}\left(gt_{i,j}+p_{i,j}-gt_{i,j}\times p_{i,j}\right)\times \left(1+\gamma w_{i,j}\right)}$$

$Z_{\mathrm{WAC}}$ and $Z_{\mathrm{ADC}}$ calculate wBCE and wIoU, respectively, named $L_{\mathrm{wBCE}}^{1},L_{\mathrm{wIoU}}^{1}$ and $L_{\mathrm{wBCE}}^{2},L_{\mathrm{wIoU}}^{2}$. Then, we fuse two loss functions to obtain the overall loss of CODCEF, the Pixel Perception Fusion Loss (PPF):

(6) $$L_{\mathrm{PPF}}=\left(L_{\mathrm{wBCE}}^{1}+L_{\mathrm{wIoU}}^{1}\right)+\left(L_{\mathrm{wBCE}}^{2}+L_{\mathrm{wIoU}}^{2}\right)$$

where $L_{t}^{i}$ means the *t* type of loss for the result of the $i^{th}$.

## 4. Evaluation

### 4.1. Datasets

We chose COD10K [20], CAMO [18] and CHAMELEON [49] as the source of the basic dataset.

The COD10K dataset is the most comprehensive and largest data set in the COD field today. COD10K includes 5066 camouflaged objects, 3000 background, 1934 non-camouflaged objects divided into 10 super-classes, and 78 sub-classes.

The CAMO dataset with 3000 pictures has more challenging camouflage pictures, focusing on artificial camouflaged objects from the art and military field.

CHAMELEON contains 76 high-resolution pictures, which is closer to the capture conditions of the camera trap.

To accomplish the training step, we mixed the default training set of COD10K (about 6000 images) and CAMO (about 1000 images), obtaining a training set containing close to 7000 images. This training set covers a variety of targets from salient targets to difficult camouflaged targets.

For the baseline comparison, we evaluated all the methods on the test set of COD10K and CAMO. Considering that there are only dozens of pictures of chameleons, we used the entire dataset as a test set.

### 4.2. Evaluation Metrics

We selected four widely used and standard metrics to evaluate the performance of CODCEF and some existing methods, which were the mean absolute error (MAE) [52], S-measure [53], F-measure [54], and E-measure [55].

MAE is used to calculate the difference between prediction maps and the ground truth. Mathematically, it is given by:

(7) $$\mathrm{MAE}=\frac{1}{H\times W}\sum_{i=1}^{H}\sum_{j=1}^{W}\left|p_{i,j}-gt_{i,j}\right|$$

where $p_{i,j}$ and $gt_{i,j}$ are point (*i*,*j*) in the prediction maps and ground truth.

S-measure [53] evaluates models with region-aware and object-aware structural similarity; this is given by:

(8) $$S_{\alpha }=\alpha S_{o}+\left(1-\alpha \right)S_{r}$$

where $S_{o}$ represents an object-aware structural similarity measure and $S_{r}$ represents the region-aware structural similarity measure. According to [53], we set α to 0.5.

F-measure [54] is a metric that can judge structural similarity, which is given by:

(9) $$F_{\beta }=\frac{(1+\beta^{2})\mathrm{Precision}\times \mathrm{Recall}}{\beta^{2}\times \mathrm{Precision}+\mathrm{Recall}}$$

where Precision is the proportion of pixels marked as detected in the prediction map and Recall consists of the ground truth. Specifically, we set $\beta^{2}$ = 0.3.

E-measure is the Enhanced-alignment measure [55], which evaluates pixel-level matching and image-level statistics. This metric is naturally suited for assessing the overall and localized accuracy of results. It is given by:

(10) $$E_{m}=\frac{1}{H\times W}\sum_{i=1}^{H}\sum_{j=1}^{W}f\left(\xi_{FM}\left(i,j\right)\right)$$

where $\phi_{FM}\left(i,j\right)$ means the enhanced alignment matrix of point (i,j), $f\left(x\right)=\frac{1}{4}{\left(1+x\right)}^{2}$ and $\xi_{FM}$ is given by:

(11) $$\xi_{FM}=\frac{2\varphi_{GT}\circ \varphi_{FM}}{\varphi_{GT}\circ \varphi_{GT}+\varphi_{FM}\circ \varphi_{FM}}$$

In implementing the metrics above, we used an evaluation tool, CODToolbox, (Available online: https://github.com/DengPingFan/CODToolbox accessed on 23 February 2021).

### 4.3. Implementation Details

#### 4.3.1. Training Implementation

We utilized the Adam optimizer [56] with batchsize = 32 to train our network. By tuning the parameters in multiple iterations, we eventually set the learning rate to 0.0001. In Pytorch 1.9 with an RTX 2080Ti GPU, we obtained the best results in 55 training epochs. The Appendix A shows the basis for our choice of learning rate.

#### 4.3.2. Testing Implementation

We tested the model on a portable edge device, NVIDIA Jetson Nano. In the performance evaluation experiment, we input the dataset image directly into the device. In order to unify the different images, we resized all input images resolution to 352×352 and normalized them. During the evaluation of the results, we up-sampled the prediction maps to the original resolution.

### 4.4. Results and Analysis

To verify the feasibility and advantages of our method, we compared CODCEF with 10 previous methods, including FPN [34], BASNet [26], PFANet [22], CPD [25], ANet [18], CSNet [51], SINet [20], RankNet [19], and R-MGL [50]. Among those, MGL, SINet, and RankNet are the state-of-the-art methods for COD. For a fair comparison, we used the same evaluation tools from CODTOOlbox at the same output resolution to generate scores.

#### 4.4.1. Overview

It can be seen from Table 1 that CODCEF demonstrated strong competitiveness in the prediction accuracy and model size. Even if the test data set contained more significant goals, the SOD domain model still lagged behind the COD model by a large margin, indicating that the challenge of the COD task is, indeed, different than that of the traditional SOD task. To locate the object when the camouflage degree of the target is close to the limit of what the naked eye can detect, a COD model is required.

**Figure 6:**
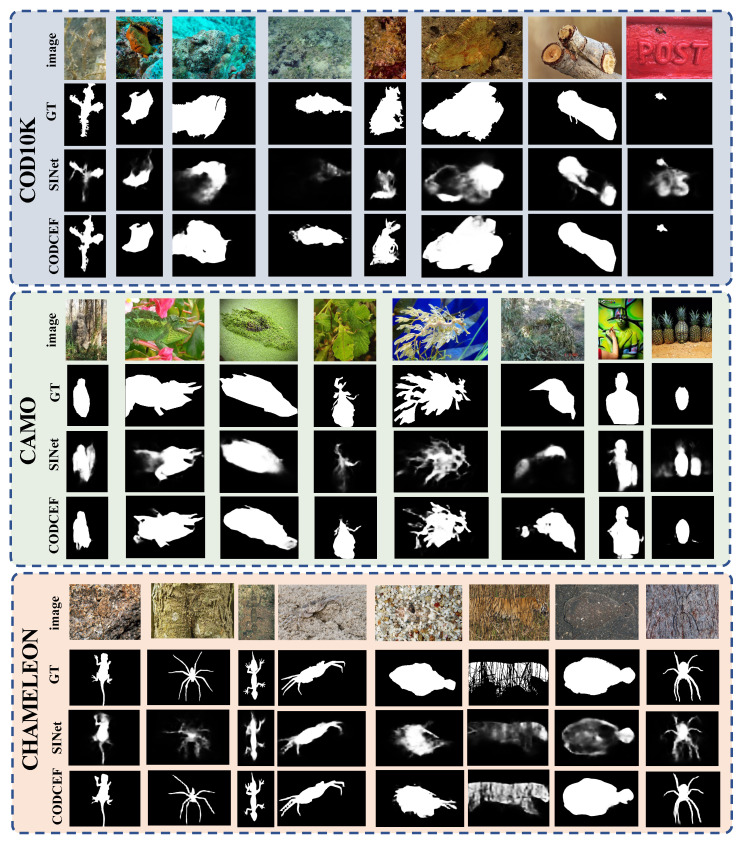
Visual comparisons of the proposed model and state-of-the-art COD algorithm SINet. Here, we show some challenging and representative scenarios: animals in nature, body painting, military camouflage, small targets, and discontinuous targets.

Compared to the earlier COD models, SINet and RankNet, CODCEF showed more powerful camouflaged target positioning capabilities and more accurate object boundary perception capabilities, outperforming them in most of metrics. A visual comparison is shown in Figure 6.

In terms of the prediction accuracy, our method is indeed slightly worse than R-MGL on $S_{\alpha }$ and $E_{m}$. However, we must note that the typical structure of R-MGL contains 444M parameters, while our method only needs half (213M), which makes our model more suitable for running in edge devices with small memory. Using selective feature fusion, CODCEF focuses on enhancing the robustness of features with a low signal-to-noise ratio without significantly increasing the complexity and size of the network. The comparison between model size and inference time is shown in Table 2.

#### 4.4.2. Performance on COD10K

COD10K, as the dataset with more than 2000 pictures and the widest coverage, can give the most representative results on COD tasks.

**Figure 7:**
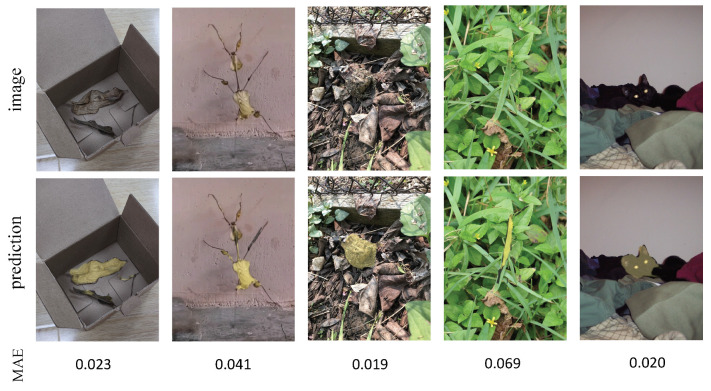
Some examples implemented on edge devices. The above examples simulate application scenarios, such as the identification of dangerous objects, portable biometric identification in the wild, and fixed-point biometric monitoring (camera traps).

As discussed above, when the target feature is highly similar to the background, the contour of the object cannot be accurately identified through the traditional feature decoding method. We show some failure cases of SINet and our corresponding predictions in the top of Figure 7, which prove that our method can produce accurate predictions with sharp object boundaries.

#### 4.4.3. Performance on CAMO

Table 1 shows that CAMO was the most challenging test dataset, due to the large proportion of artificial camouflaged objects, for example body painting and military camouflage. Therefore, even the state-of-the-art camouflaged object detection model was unable to obtain acceptable results, which is shown in the middle of Figure 7. Given such a rigorous data set, we verified the robustness of CODCEF in the case of extremely low feature signal-to-noise ratios. We also noticed that, for certain artificial camouflage objects, CODCEF had issues in determining the confusing part of the image.

#### 4.4.4. Performance on CHAMELEON

Compared with the previous datasets, the significance of the target in CHAMELEON was the closest to that of the SOD task. In CHAMELEON, CODCEF outperformed all 10 SOD and COD models in four metrics, which proves that our model not only dealt with difficult camouflage images but also had versatility for ordinary salient target images.

### 4.5. Ablation Study

In this section, we validate the effectiveness of our method by replacing or removing the structure or loss function we proposed.

#### 4.5.1. Structure Ablation

CODCEF can be divided into four main components, WAC (Section 3.2), ADC (Section 3.3), RF (Figure 4), and FPD (Section 3.4). For the first two, we evaluated the output of WAC to prove the necessity of the cascade structure. For the latter two, we replaced RF with a 1×1 convolutional layer, and replaced PFD with the baseline structure proposed in [25]. The evaluation results are shown in Table 3. Comparing all the listed component combinations, the original structure of CODCEF performed best on COD10K.

- ADC ablation: The removal of an ADC is equivalent to abandoning the cascading structure, which directly leads to the lack of boundary refinement in the prediction results. The experimental results demonstrated a significant image degradation after removing the ADC, $S_{\alpha }$, which was more sensitive to the details of the results. In other words, the depth of the model introduced by the ADC did not produce a significant degradation in the prediction accuracy. Compared with F3Net, our structure can accommodate more sub-decoders to provide more visual perception capabilities.
- RF ablation: Our motivation for using RF was to reduce the dependence on deep backbone networks. After replacing RF with a common convolutional layer, ResNet50 could not extract the basic features of the available level, which led to the rapid degradation of the prediction results. This shows that the introduction of RF effectively enhanced the features extracted by the encoder.
- FPD ablation: Compared with the traditional decoder [25] used in SINet, FPD had a stronger ability to improve the signal-to-noise ratio, which is extremely important for COD tasks. The experimental results demonstrated the excellent performance of FPD.

#### 4.5.2. Loss Function Ablation

To further investigate the performance of PPF, we chose the binary cross entropy (BCE), which is widely used in the SOD field as a benchmark for comparison. As seen in Table 4, when BCE was used as the loss function, the model obtained a significant performance degradation.

## 5. Real Environment Experiment

In order to verify the feasibility of deploying our model in a real environment, we designed a portable image acquisition and detection device based on Jetson nano.

### 5.1. Experiment Implement

We implemented CODCEF on the NVIDIA Jetson Nano with a Raspberry Pi Camera v2 (IMX219 with a quarter-inch aperture) as the image acquisition device and a 7.5 W mobile power supply as the power source, using the model trained in Section 4. In the wild and indoor environments, we photographed 37 camouflaged targets (27 images of camouflaged creatures and 10 other images) and generated segmentation results in real time.

### 5.2. Result and Analysis

Compared with those in the datasets, the images taken in the real environment contained more varied target types, and the image quality was limited by the performance of the sensor and the illumination. We attempted to simulate non-invasive biological image acquisition (camera trap) in biological research in several different field locations and photographed targets that were less relevant to the training data. As can be seen from Figure 7, our method showed a stable segmentation ability in dealing with animals that were integrated into the background environment. CODCEF also showed considerable generalization ability for targets that are rarely seen in the training samples. For example, leaves are generally used as background filtering in training data; however, in the example on the left in Figure 7, CODCEF accurately recognized it as a camouflage target.

## 6. Conclusions

In this paper, we proposed a new framework for camouflaged object detection, namely CODCEF. CODCEF consists of two relatively independent and cascaded perceptual modules. Compared with traditional single encoder–decoder structures, our architecture showed stronger detection accuracy and robustness. To undertake the feature decoding task of CODCEF, we used a Cross Feature Module to build a Feedback Partial Decoder (FPD), which effectively reduced misleading information brought about by camouflage images. In addition, we proposed the novel loss function Pixel Perception Fusion Loss (PPF) to mitigate possible target edges.

The experiments showed that CODCEF achieved state-of-the-art performance on three benchmark datasets of camouflaged object detection on four evaluation metrics. Ablation studies on structures and loss functions demonstrated the superiority and reliability of our method. The main limitation of CODCEF lies in the lack of generalization ability caused by the training samples. For target types that do not appear in large-scale training samples, such as human military camouflage, CODCEF’s prediction accuracy is limited. In future work, we will further introduce semi-supervised learning to deal with the lack of target training data in certain fields.

## Figures and Tables

**Table 1:** Performance comparison with 10 representative data sets from the SOD or COD field. ↑ indicates that the higher the better, and vice versa. As ANet-SRM [18] has no public original code, we directly used the results obtained on the CAMO dataset in the original text. We marked the best two scores of every metric in red and blue, respectively.

Models	CHAMELEON [49]				COD10K [20]				CAMO [18]			
	M↓	$S_{\alpha }↑$	$F_{\beta }↑$	$E_{m}↑$	M↓	$S_{\alpha }↑$	$F_{\beta }↑$	$E_{m}↑$	M↓	$S_{\alpha }↑$	$F_{\beta }↑$	$E_{m}↑$
FPN${}^{2017}$ [34]	0.075	0.794	0.590	0.783	0.075	0.697	0.411	0.691	0.131	0.684	0.483	0.677
BASNet${}^{2019}$ [26]	0.118	0.687	0.474	0.721	0.105	0.634	0.365	0.678	0.159	0.618	0.413	0.661
PFANet${}^{2019}$ [22]	0.144	0.679	0.378	0.648	0.128	0.636	0.286	0.618	0.172	0.659	0.391	0.622
CPD${}^{2019}$ [25]	0.052	0.853	0.706	0.866	0.059	0.747	0.508	0.770	0.115	0.726	0.550	0.729
CSNet${}^{2019}$ [51]	0.051	0.819	0.759	0.859	0.048	0.745	0.615	0.808	0.106	0.704	0.633	0.753
F3Net${}^{2020}$ [27]	0.047	0.848	0.770	0.894	0.051	0.739	0.593	0.795	0.109	0.711	0.616	0.741
ANet${}^{2019}$ [18]	-	-	-	-	-	-	-	-	0.126	0.682	0.484	0.685
SINet${}^{2020}$ [20]	0.044	0.869	0.740	0.891	0.051	0.771	0.551	0.806	0.100	0.751	0.606	0.771
RankNet${}^{2021}$ [19]	0.046	0.842	0.794	0.896	0.045	0.760	0.658	0.831	0.105	0.708	0.645	0.755
R-MGL${}^{2021}$ [50]	0.030	0.893	0.813	0.923	0.035	0.814	0.666	0.865	0.088	0.775	0.673	0.847
CODCEF(Ours)	0.030	0.875	0.825	0.932	0.043	0.766	0.667	0.854	0.092	0.736	0.685	0.797

**Table 2:** Comparison of the model accuracy and complexity. The infer time is measured on an RTX 2080Ti.

Model	Params	Infer Time	CHAMELEON		COD10K		CAMO	
			$E_{m}↑$	$F_{\beta }↑$	$E_{m}↑$	$F_{\beta }↑$	$E_{m}↑$	$F_{\beta }↑$
SINet	198M	32 ms	0.891	0.740	0.806	0.551	0.771	0.606
R-MGL	444M	48 ms	0.923	0.813	0.865	0.666	0.847	0.673
CODCEF	212M	37 ms	0.932	0.825	0.854	0.667	0.802	0.685

**Table 3:** The ablation study results of structure on COD10K. Note that after replacing RF with a simple single-layer 1× 1 convolutional layer, the loss function cannot converge to an acceptable range. Thus, we have surpassed the best result on each metric.

Structure				COD10K			
WAC	ADC	RF	FPD	M↓	$S_{\alpha }↑$	$F_{\beta }↑$	$E_{m}↑$
✓		✓	✓	0.048	0.747	0.573	0.845
✓	✓		✓	0.301	0.446	0.195	0.428
✓	✓	✓		0.085	0.649	0.542	0.799
✓	✓	✓	✓	**0.043**	**0.766**	**0.667**	**0.854**

**Table 4:** The ablation study results of the loss function on COD10K.

Loss	COD10K			
	M↓	$S_{\alpha }↑$	$F_{\beta }↑$	$E_{m}↑$
PPF (ours)	**0.043**	**0.766**	**0.667**	**0.854**
BCE	0.049	0.749	0.558	0.824
drop (%)	8.8	2.8	9.2	3.5

**Figure A1:**
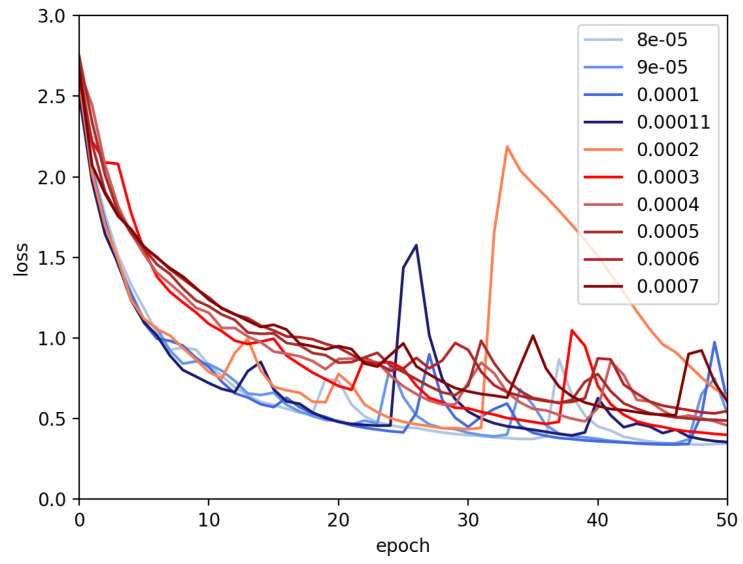
The convergence of the loss function while training under different learning rates. Other implementation details are inherited from Section 4.3.

## Data Availability

The datasets analyzed in this study are available through their respective repository pages. COD10K [20] is at http://dpfan.net/Camouflage/. CAMO [18] is at https://sites.google.com/view/ltnghia/data. CHAMELEON is at http://kgwisc.aei.polsl.pl/index.php/en/dataset/63-animal-camouflage-analysis (accessed on 15 January 2021).

## Appendix A. Hyperparameter Selection

As shown in Figure A1, we tested different learning rates and recorded the training loss. We chose the most stable learning rate, 0.0001.
